# Green Coffee Extract Improves Cardiometabolic Parameters and Modulates Gut Microbiota in High-Fat-Diet-Fed ApoE^-/-^ Mice

**DOI:** 10.3390/nu11030497

**Published:** 2019-02-27

**Authors:** Erika Caro-Gómez, Jelver A. Sierra, Juan S. Escobar, Rafael Álvarez-Quintero, Mauricio Naranjo, Sonia Medina, Eliana P. Velásquez-Mejía, Jorge H. Tabares-Guevara, Julio C. Jaramillo, Yudy M. León-Varela, Katalina Muñoz-Durango, José R. Ramírez-Pineda

**Affiliations:** 1 Grupo Inmunomodulación—GIM, Universidad de Antioquia. Calle 70 No. 52-21, 050010 Medellín, Colombia; erikabril@gmail.com (E.C.-G.); jorgetabare@gmail.com (J.H.T.-G.); jcjara05@gmail.com (J.C.J.); yudyleon@gmail.com (Y.M.L.-V.); 2 Vidarium–Nutrition, Health and Wellness Research Center, Grupo Empresarial Nutresa. Calle 8 Sur No. 50-67, 050023 Medellín, Colombia; jasierra@serviciosnutresa.com (J.A.S.); jsescobar@serviciosnutresa.com (J.S.E.); epvelasquez@serviciosnutresa.com (E.P.V.-M.); 3 Grupo de Investigación en Sustancias Bioactivas, Facultad de Ciencias Farmacéuticas y Alimentarias, Universidad de Antioquia. Calle 70 No. 52-21, 050010 Medellín, Colombia; ralvarezq@ces.edu.co; 4 Colcafé Research Coffee Group, Industria Colombiana de Café S.A.S. Calle 8 Sur No. 50-19, 050023 Medellín, Colombia; mnaranjo@colcafe.com.co; 5 Facultad de Ingeniería, Corporación Universitaria Lasallista, Carrera 51 N°118Sur-57, 055440 Caldas, Colombia; soniamedes@gmail.com

**Keywords:** green coffee, atherosclerosis, cardiometabolic syndrome, NAFLD, high fat diet, immune system, gut dysbiosis

## Abstract

Chlorogenic acids (CGA) are the most abundant phenolic compounds in green coffee beans and in the human diet and have been suggested to mitigate several cardiometabolic risk factors. Here, we aimed to evaluate the effect of a water-based standardized green coffee extract (GCE) on cardiometabolic parameters in ApoE^-/-^ mice and to explore the potential underlying mechanisms. Mice were fed an atherogenic diet without (vehicle) or with GCE by gavage (equivalent to 220 mg/kg of CGA) for 14 weeks. We assessed several metabolic, pathological, and inflammatory parameters and inferred gut microbiota composition, diversity, and functional potential. Although GCE did not reduce atherosclerotic lesion progression or plasma lipid levels, it induced important favorable changes. Specifically, improved metabolic parameters, including fasting glucose, insulin resistance, serum leptin, urinary catecholamines, and liver triglycerides, were observed. These changes were accompanied by reduced weight gain, decreased adiposity, lower inflammatory infiltrate in adipose tissue, and protection against liver damage. Interestingly, GCE also modulated hepatic IL-6 and total serum IgM and induced shifts in gut microbiota. Altogether, our results reveal the cooccurrence of these beneficial cardiometabolic effects in response to GCE in the same experimental model and suggest potential mediators and pathways involved.

## 1. Introduction

Industrialization has led to significant changes in lifestyle and eating behaviors [1,2,3]. Epidemiological studies have linked cardiometabolic syndrome (CMS) to habits that are common in Westernized populations, such as hypercaloric diets and sedentary lifestyles, and have anticipated a significant increase in the incidence of CMS in the decades to come [4]. CMS refers to the cooccurrence of several interrelated conditions that share primary mediators, mechanisms, and pathways, including insulin resistance/hyperglycemia, visceral adiposity/obesity, atherogenic dyslipidemia, and hypertension [5]. These alterations, in turn, increase the risk for type 2 diabetes, atherosclerotic cardiovascular diseases (CVDs), and nonalcoholic fatty liver disease (NAFLD) [6,7]. Studies have identified chronic inflammation, triggered by metabolic dysfunction and gut microbiota dysbiosis, as an underlying cause of the onset and progression of CMS. Alterations in energy homeostasis and metabolic processes, as well as interferences with pro-/anti-inflammatory pathways, have been proposed as common mechanisms linking hypercaloric diets and dysbiosis to CMS [8,9]. Our current understanding suggests that treating inflammation and microbiota alterations might represent an opportunity for CMS management [10,11].

There is growing interest in finding natural compounds that might help treat CMS. The most cost-effective measure to control CMS is through the modification of dietary patterns and the adoption of a healthy lifestyle, including personalized diets, physical activity, and cognitive-behavioral therapy [12]. Interestingly, a variety of studies have demonstrated the potential of dietary polyphenols for controlling CMS [13], implying that dietary interventions might play a role in the amelioration of CMS-related complications. Phenolic compounds account for approximately 50% of the daily intake of polyphenols, and chlorogenic acids (CGA) are the most abundant phenolic compounds in the human diet, with coffee and fruits representing the main sources of phenolic compounds [14,15,16]. Epidemiological and observational studies have found that mortality rates related to CMS are inversely associated with coffee consumption, and improved liver health and reduced diabetes risk have also been reported. However, the mechanisms and molecular targets related to such effects remain elusive [17,18,19]. It has been hypothesized that the beneficial effects of coffee on CMS are largely related to its antioxidant activity and to the improvement of cellular processes, including insulin sensitivity and the modulation of other metabolism-related pathways, but the immunomodulatory activity and prebiotic properties of coffee have been less explored.

Most studies showing how coffee intake can improve cardiometabolic health have used roasted coffee; however, the roasting process is known to alter the bioactive profile of the coffee [20]. Green (unroasted) coffee beans contain high amounts of bioactive substances, mainly CGAs, caffeine, and soluble fiber (mainly galactomannans and arabinogalactans [21]). Standardized green coffee extracts (GCEs) that contain bioactive compounds at high concentrations are a feasible option and are known to retain the beneficial effects that coffee has on CMS, including improvements in glucose and lipid metabolism and anti-obesity effects [22,23,24,25,26]. Recent studies have also suggested that bioactive compounds in GCE can be biotransformed by, and even modulate, the gut microbiota [21,27,28,29], which is an interesting finding since dysbiosis has been linked to obesity and cardiometabolic disease [30,31,32].

Apolipoprotein E-deficient (ApoE^-/-^) mice represent a good model to study cardiovascular and lifestyle-related metabolic diseases [33,34]. ApoE deficiency induces severe hyperlipidemia, which is accelerated in animals that are fed an atherogenic/obesogenic high-fat diet (HFD); ApoE deficiency is associated not only with atherosclerosis but also with most features of CMS [5,35]. In this study, we aimed to investigate the effects of oral treatment with a water-based GCE on atherosclerosis and a set of CMS features that are known to increase cardiovascular risk using HFD-fed ApoE^-/-^ mice as a model. Atherosclerotic lesions, body weight, adiposity, NAFLD, glucose and other indicators related to metabolic homeostasis, as well as relevant immune-metabolic markers and gut microbiota composition, were assessed. 

## 2. Materials and Methods

### 2.1. Reagents

Paraformaldehyde, sucrose, Oil Red O (ORO), Sirius red, hematoxylin, pyridine, N,O-bis(trimethylsilyl)trifluoroacetamide (BSTFA), ammonium formate, ammonium acetate, dopamine, dopamine-d4, 3-methoxytyramine, 3-methoxytyramine-d4, norepinephrine, norepinephrine-d6, epinephrine, epinephrine-d6, normetanephrine, normetanephrine-d3, metanephrine, metanephrine-d3, nonadecanoic acid C19, triglycerides, and cholesterol standards were purchased from Sigma-Aldrich (St. Louis, MO, USA). Methanol, chloroform, hexane-acetonitrile, formic acid, and hydrochloric acid were acquired from Merck (Kenilworth, NJ, USA). Water was treated with a Purelab^®^ ultra analytic purification system from ELGA LabWater (High Wycombe, UK). PBS was obtained from Gibco (New York, NY, USA).

### 2.2. Green Coffee Bean Extract (GCE)

Spray-dried GCE was produced by Colcafé S.A.S from *Coffea canephora* var. *robusta* beans using hot water as the extract solvent and was kept at room temperature in laminated vacuum-sealed packaging until use. Total CGA and caffeine content were measured with standard HPLC procedures [36,37]. Fatty acids, cholesterol, total carbohydrates, total dietary fiber, total protein, minerals, ash, acrylamide, aflatoxin, zearalenone, and ochratoxin were analyzed by Covance Inc. (Princeton, NJ, USA).

### 2.3. Mice, Treatments, and Sample Collection

**Figure 1:**
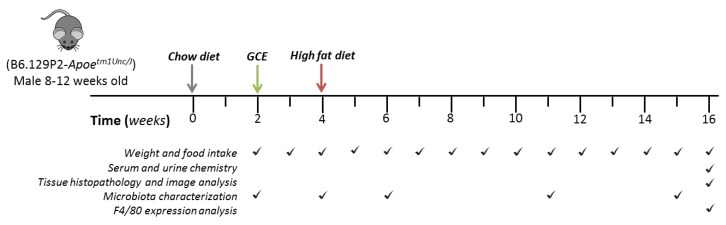
Study design and sampling scheme.

ApoE^-/-^ mice obtained from Jackson Laboratories (Bar Harbor, ME, USA) were bred and housed at 22 ± 1 °C under a 12 h light/dark cycle with free access to food and water and were maintained under SPF conditions at the Universidad de Antioquia animal facility. The mice were housed in cages with up to 5 animals and acclimated to their environment prior to the experiment. Mice were then randomly allocated to the vehicle (*n* = 10) group or the GCE (*n* = 14) group. The first four weeks of the experiment mice were fed a regular chow diet (Laboratory Rodent Diet 5001, Labdiet, St. Louis, MO) and then shifted to an HFD containing 42% kcal from fat (Teklad Custom diet TD.88137 ENVIGO, Tampa, FL, USA). Each animal received GCE from the beginning of the second week while still on chow diet, either GCE (equivalent to 220 mg/kg of CGA) or sterile water by oral gavage (200 µL/mouse) three times a week, until the end of the experiment. During the experiment, food intake and body weight were recorded weekly. The selected CGA dose was derived from preliminary studies performed on wild-type C57BL/6 mice, aimed towards estimating the amount of GCE tolerated by the animals containing the highest dose of CGAs. Animals were examined daily for changes in behavior, drinking/eating patterns, appearance, and weight loss; the selected dose was well tolerated. At the end of the experiment (Week 16), the animals were fasted overnight (12–14 h) and sacrificed (Figure 1). Serum and urine samples were collected and stored at −80 °C until further analysis. Following the blood collection, hearts were dissected after in situ perfusion with PBS (Figure 1), and the epididymal white adipose tissue (WAT), perirenal WAT, and liver were removed, rinsed with PBS, and weighed. All experiments were approved by the Institutional Animal Care and Use Committee (Meeting 92, 30 January 2015) of the Universidad de Antioquia, Medellin, Colombia.

### 2.4. Atheroprotective Effect Assessment

Hearts were fixed using buffered 4% paraformaldehyde for 48 h, immersed in three changes of 30% sucrose solutions for 24 h each, embedded in Shandon Cryomatrix™ (Thermo Scientific Inc., Waltham, MA, USA) and then frozen at −20 °C. Cryo-sections (Leica Microsystems, Wetzlar, Germany) were obtained as previously described [38], and the areas of atherosclerotic lesions in the aortic sinus were quantified in 8-μm-thick transverse sections. Averages of the total atherosclerotic plaque area and lipid deposition were calculated from serial Oil Red O/hematoxylin stained sections and were expressed as μm^2^ and as the sum of red pixels, respectively. Macrophage and T cell infiltration were analyzed by immunofluorescence and reported as the sum of red pixels. Briefly, aortic sinus sections were acetone-fixed, treated with universal antigen retrieval solution (Innovex Biosciences Inc., Richmond, CA, USA), and incubated with macrophage- or T cell-specific monoclonal antibodies (anti-mouse CD68, clone FA-11, or anti-CD3, clone KT3, respectively; Bio-Rad Laboratories Inc. Hercules, CA, USA). A secondary goat anti-rat IgG antibody labeled with Alexa 594 (Thermo Fisher Scientific Inc., Waltham, MA, USA) was used, and sections were mounted using VECTASHIELD™ Antifade Mounting Medium with DAPI (Vector Laboratories Inc., Burlingame, CA, USA). Images were obtained with a Zeiss Axio Scope.A1 microscope (Carl Zeiss, Oberkochen, Germany) and analyzed using the NIS Elements BR image analysis software (Nikon, Tokyo, Japan). The results are reported as the mean of 6–8 sections per animal.

### 2.5. Blood and Urine Biochemistry

Serum levels of glucose, triglycerides, total cholesterol, high-density lipoprotein cholesterol (HDL-C), low-density lipoprotein cholesterol (LDL-C), alanine aminotransferase (ALT), aspartate aminotransferase (AST), alkaline phosphatase (ALP), creatinine, α-amylase, and lactate dehydrogenase (LDH) were determined using colorimetric kits (Biosystems S.A., Barcelona, Spain). Serum levels of insulin (Crystal Chem Inc., Downers Grove, IL, USA), insulin-like growth factor 1 (IGF-1) (Abcam plc, Cambridge, UK), leptin, adiponectin, and total IgM and IgG (R&D Systems, Minneapolis, MN, USA) were measured with ELISA kits. The homeostatic model assessment (HOMA) method [39] was used to calculate insulin resistance (HOMA-IR) and β-cell function (HOMA-B). HOMA-IR was calculated as (fasting insulin × fasting glucose)/22.5, and HOMA-B was calculated as (20 × fasting insulin)/fasting glucose-3.5, using mmol/L for glucose and μU/mL for insulin. Insulin sensitivity was estimated using the quantitative insulin sensitivity check index (QUICKI) [40], calculated as 1/(log(fasting insulin)+log(fasting glucose)), using mg/dL units for glucose and μU/mL for insulin.

Extractions of catecholamines and metanephrines from urine samples were performed using an application note from Waters [41]. Two hundred microliters of urine samples were pretreated with 25 µL of 1 N HCl, 25 µL of deuterated internal standards, and 0.5 mL of 0.5 M NH_4_CH_3_COO. The mass spectrometer was operated in positive electrospray ionization mode (ESI^+^) using selected reaction monitoring (SRM) with quantification and confirmation transitions for each analyte (Table S1). Nitrogen was used as the desolvation gas at a 650 Lh^−1^ flow rate with a 200 °C desolvation temperature, a 3.10 kV capillary voltage, and a 19 V cone voltage. Data acquisition and quantification were performed using Waters MassLynx V4.1. (Milford, MA, USA).

### 2.6. Liver and Adipose Tissue Analyses

Subsequently, 10-µm-thick sections of frozen epididymal fat pads were obtained as described above. Overnight formaldehyde-vapor-fixed sections were stained with hematoxylin; adipocyte size (μm^2^) and diameter (μm) were analyzed using the NIS Elements BR image analysis software (Nikon, Tokyo, Japan). First, images were converted into binary format using the automated measurement feature, and the threshold was adjusted to match the contour of the objects. Objects touching the edges, not clearly defined, or <300 μm^2^ were excluded. The results are reported as the mean of 2–3 sections per animal. Liver and epididymal WAT were preserved in RNAlater (Qiagen Inc., Germantown, MD, USA) and stored at −80 °C until use. Total RNA was isolated using the RNeasy™ Mini Kit (Qiagen, Germany). Tissue homogenates were centrifuged (twice at 17,800× *g*, 10 min, 4 °C), and the lipid layer was removed before phase separation. cDNA was synthesized from 100 ng of RNA using a Revert Aid H Minus First Strand cDNA synthesis kit (Thermo Scientific Inc., Waltham, MA). F4/80 was amplified by quantitative PCR (qPCR) using Applied Biosystems™ TaqMan® Gene Expression Assays (Foster, CA, USA) on a LightCycler™ 96 (Roche, Penzberg, Germany). Relative mRNA levels were calculated for each animal using the ΔCt method and were normalized to *hypoxanthine phosphoribosyl transferase* (*HPRT*) as a reference gene. Subsequently, 10-µm-thick sections of frozen livers were obtained and stained with Oil Red O/hematoxylin or picrosirius red for lipid and collagen deposition analysis, respectively. The histological change assessment and score scales are presented in Table S2. Images were obtained and analyzed as described above. Cytokine quantification in liver samples was performed as follows: 100 mg of tissue was homogenized in 500 µL of cOmplete™ Protease Inhibitor Cocktail (Roche Applied Science, Penzberg, Germany) using the BeadBlaster™ Homogenizer (Benchmark Scientific, NJ, USA). Supernatants were collected after centrifugation (1500× *g*, 5 min, 4 °C) and stored at −80 °C until use. IL-6, TNF-α, and IL-10 in liver homogenate supernatants were measured using DuoSet™ ELISA kits (R&D Systems, Minneapolis, MN, USA). Total hepatic lipid fractions were extracted from 60–70 mg of lyophilized samples supplemented with BHT (50 μg/mL) + DHPG + C19 using 900 μL of chloroform/hexane (3:7). Tissue disruption was achieved by 2 min of homogenization with BeadBlaster™ Homogenizer (Benchmark Scientific, NJ, USA), centrifugation at 1500× *g*, and sonication for 30 min at 60 Hz. Samples were then allowed to stand for 6 h at 10 °C, followed by sonication for 10 min and centrifugation at 16,000× *g* for 10 min at 4 °C. Supernatants were split and evaporated for GC-MS/GC-FID analysis of free fatty acids (100 μL), cholesterol (100 μL), and triglycerides (500 μL) using an Agilent 7890 GC (Wilmington, DE) equipped with a 5975C mass selective detector (MSD) and a flame ionization detector (FID). Conditions for each analysis are presented in Table S3.

### 2.7. Gut Microbiota Analysis

Fecal samples were collected from 23 animals at five time points (Figure 1) and kept at −80 °C until DNA extraction. Total microbial DNA was extracted with the MoBio PowerSoil® DNA Isolation Kit cat# 12888 (Carlsbad, CA, USA). DNA concentrations were obtained with a Synergy HT Microplate Reader (Bio-Tek Instruments, Winooski, VT, USA). We also included positive (mock community; Zymo Research cat# D6306) and negative (elution buffers and ultrapure water) controls to estimate sequencing error rates and contamination introduced during sample manipulation, respectively. DNA from samples and controls were sent to the University of Michigan Medical School Host Microbiome Initiative (Ann Arbor, MI, USA), where the V4 region of the 16S rRNA gene was amplified with primers F515 and R806. Multiplex PCR was performed with dual indices [42], and the library was normalized using the SequalPrep Normalization Plate Kit (Life Technologies, cat# A10510-01). The concentration of the pooled samples was determined using the Kapa Biosystems Library Quantification kit for Illumina platforms (cat# KK4824). The sizes of the amplicons in the library were determined using the Agilent Bioanalyzer High Sensitivity DNA analysis kit (cat# 5067-4626). The final library consisted of equal molar amounts from each of the plates, normalized to the pooled plate at the lowest concentration (8.0 nM). Finally, the pooled library was sequenced using the Illumina MiSeq sequencing platform with the reagent kit V2. Raw sequences were deposited in the NCBI’s Short Read Archive (SRA) under BioProject PRJNA521264. Raw 16S rRNA gene sequences were processed using Mothur v.1.39 following the MiSeq standard operating procedure [42]. 

### 2.8. Statistical Analysis

Data are reported as the mean ± 95% CI unless otherwise specified. Assumptions of normal distribution of residuals and homogeneity of variances were checked using D’Agostino-Pearson omnibus and Bartlett tests, respectively. GCE and vehicle groups were compared with unpaired Student’s t-tests; when assumptions were violated, data were log-transformed. Comparisons involving discrete data were performed using Mann–Whitney U tests. Weight gain over time was compared between groups using a two-way repeated measures ANOVA; we used the Sidak test to adjust for multiple comparisons. GraphPad Prism® version 8.01 for Windows (GraphPad Software Inc., San Diego, CA, USA) was used for analyses. Gut microbiota diversity and composition were assessed by quantifying similarities based on alpha and beta diversities. For alpha diversity, we calculated the number of observed OTUs of each sample and tested whether this index differed between the GCE and vehicle animals using linear mixed-effects models with REML optimization [43]. Treatment was considered a fixed factor, and time and the cage in which animals were maintained were considered random (nested) factors. Beta diversity was assessed using weighted and unweighted UniFrac distances on rarefied read counts (2500 reads/sample). We tested for differences in beta diversity between treatments, sampling times, and mouse cages using permutational multivariate analysis of variance (PERMANOVA). In this case, permutations were restricted within the cages in which animals were maintained, using the strata argument in the adonis function of R [44]. We also used the rarefied OTU table to identify microbes that were statistically associated with the chow diet (Week 2, where no supplementation was still provided to animals), the HFD, and GCE (Week 15), using linear discriminant analysis effect size (LEfSe) [45]. LEfSe results were adjusted for multiple testing using the q value package of R [46]. For stringency, an OTU was retained if it had a *p*-value < 0.05, a *q*-value < 0.10, and a (log10) LDA score ≥ 3. Median relative abundances of discriminant OTUs were calculated, and OTUs with the same taxonomic classification were added and plotted. Finally, we performed a metagenomic inference based on 16S rRNA gene sequences using Tax4Fun2 [47] on the rarefied OTU table. For this, we blasted our sequences to the BLAST non-redundant database (Ref100NR) and calculated relative abundances of the Kyoto Encyclopedia of Genes and Genomes (KEGG) functional and pathway profiles of each sample at Week 15. LEfSe and q value were employed to test for statistically over-represented KEGG pathways in mice treated with vehicle or GCE. Due to the large amount of OTUs unused in the metagenomic prediction, LDA scores ≥ 2 were considered significant.

## 3. Results

### 3.1. GCE Chemical Characterization

Coffee extracts are complex mixtures containing a large number of components, and their quality depends not only on the contents of healthy compounds but also on contamination with toxins from toxigenic fungi [48], which are natural contaminants of coffee that are a public health concern [49]. A thorough physicochemical characterization illustrating the composition and lack of contamination with mycotoxins of our GCE is presented in Table 1.

### 3.2. GCE Treatment is not Atheroprotective but Improves Metabolic Dysregulation and Insulin Resistance in HFD-Fed ApoE^-/-^ Mice

**Figure 2:**
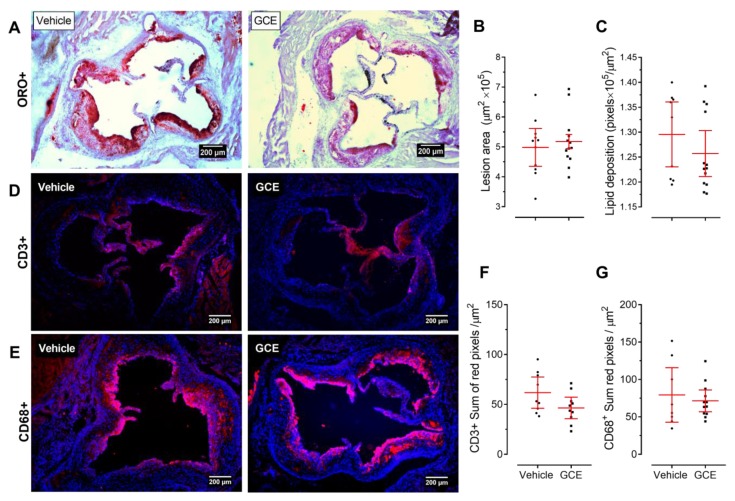
Green coffee extract (GCE) is not atheroprotective in ApoE^-/-^ mice. Animals were fed an High-fat diet and treated orally with GCE (equivalent to 220 mg/kg of chlorogenic acids, three times a week) for 12 weeks, as shown in Figure 1. Vehicle (sterile water)-treated mice were used as negative controls. (**A**) Representative histopathological images of the valve area of the aortic sinus stained with Oil Red O/hematoxylin. (**B**) Area of atherosclerotic lesions and (**C**) plaque lipid content were assessed by Oil Red O staining (ORO^+^ 1 × 10^5^/μm^2^). Aortic root sections were immunofluorescently stained with (**D**) anti-CD3 (upper panel) and (**E**) anti-CD68 (lower panel) monoclonal antibodies; representative merged images (4′,6-diamidino-2-phenylindole DAPI + Alexa 594 positive staining) are shown. In (**F**,**G**) the corresponding quantitative analyses of T cell infiltration or macrophage within the atherosclerotic plaque. Each point represents the average measurements (out of 6–8 sections) per animal (D–G). Values are expressed as the mean ± 95% confidence intervals. Unpaired t-tests did not show statistically significant differences between groups.

Based on previous work supporting favorable cardiometabolic effects of GCE and CGA administration [25,50,51,52,53], we first investigated whether our standardized GCE affected atherosclerosis development in dyslipidemic ApoE^-/-^ mice. As shown in Figure 2A, the GCE treatment did not reduce the area of atherosclerotic lesions in the aortic sinus. No differences in plaque size or lipid content were observed between the two experimental groups (Figure 2B,C), suggesting that GCE treatment at the administered dose did not attenuate or exacerbate plaque formation. Consistent with the lack of atheroprotective effect of GCE in ApoE^-/-^ mice, in immunofluorescence staining we did not observe differences in the macrophage or T cell infiltrates between treated and control animals (Figure 2D–G). Similarly, the serum lipid profile remained unchanged after GCE administration (Table 2). HFD-fed ApoE^-/-^ mice develop not only dyslipidemia and atheromatous lesions in the vascular tree but also many metabolic disturbances associated with CMS [33,34,54]. Interestingly, despite the lack of atheroprotective effects, the GCE treatment had a notable beneficial effect on glucose metabolism, as indicated by significant reductions in fasting glucose levels in GCE-treated animals compared to the same parameters in the controls, although fasting insulin levels were not affected (Table 2). Consequently, GCE-treated mice consistently showed decreased insulin resistance and improved insulin sensitivity, with no changes in β-cell function, as assessed by the HOMA and QUICKI indices (Table 2).

### 3.3. Treatment of ApoE^-/-^ Mice with GCE Attenuates HFD-Induced Weight Gain and Adiposity Increase

**Figure 3:**
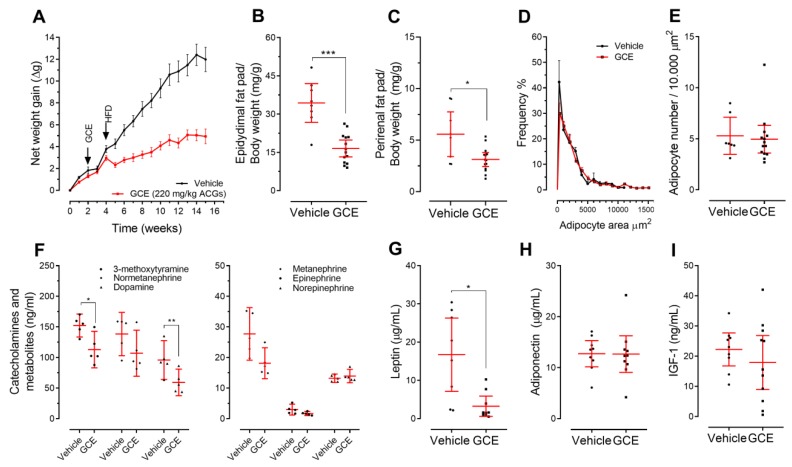
Green coffee extract (GCE) modulates body weight gain and reduces adiposity in ApoE^-/-^ high-fat diet-fed mice. The weights of the mice were recorded weekly for 16 weeks. (**A**) The average net weight gain per treatment is presented as the mean ± SEM. (**B**) Epididymal and (**C**) perirenal fat pad weights were normalized against total body weight. (**D**) Adipocyte area and (**E**) adipocyte number were quantified in epididymal fat sections. (**F**) Urinary levels of catecholamines and metanephrines were determined by high performance liquid chromatography–mass spectrometry (HPLC-MS). Serum levels of (**G**) leptin, (**H**) adiponectin and (**I**) Insulin-like growth factor 1 (IGF-1) were measured by enzyme-linked immunosorbent assay (ELISA). The results are shown as the mean ± 95% confidence intervals, and *p*-values from unpaired two-tailed t-tests are expressed as follows: * *p* < 0.05; ** *p* < 0.01; *** *p* < 0.001.

**Figure 4:**
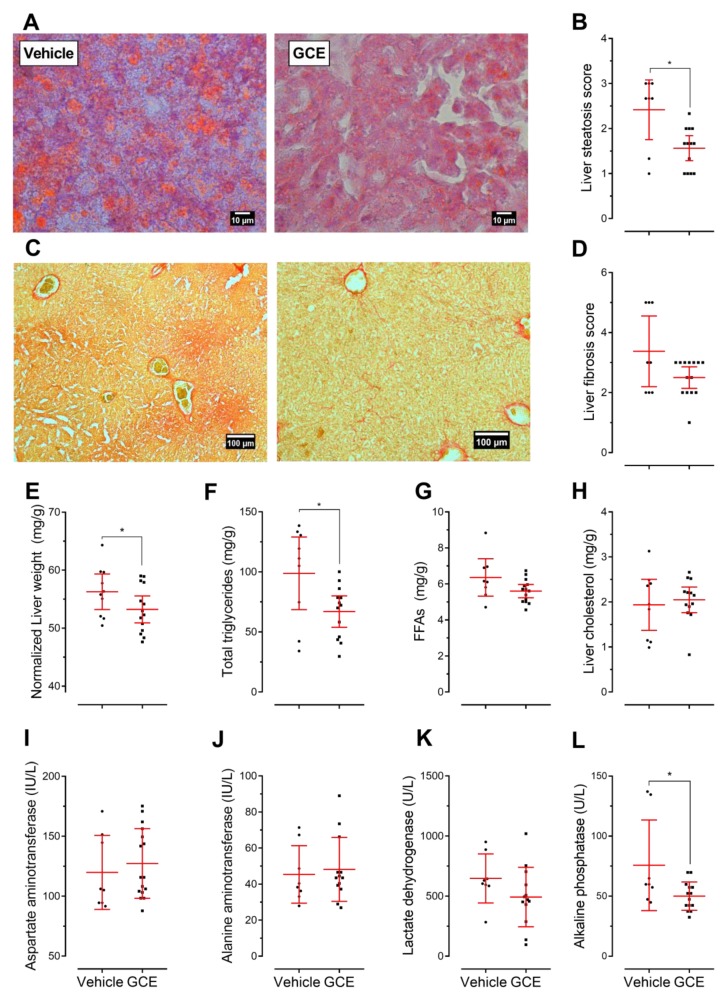
Green coffee extract (GCE) treatment ameliorated lipid accumulation and fibrosis in the livers of ApoE^-/-^ high-fat diet-fed mice. (**A**,**C**) Representative microphotographs from liver sections stained with Oil Red O/hematoxylin and the steatosis assessment of these sections. (**B**,**D**) Picrosirius red stained sections and liver fibrosis assessed as collagen deposition. Liver steatosis and fibrosis were scored according to the criteria described in supplementary Table S2. (**E**) Liver weight normalized to total body weight. (**F**–**H**) Total liver triglycerides, free fatty acids, and cholesterol contents, respectively. Markers of liver function: (**I**) aspartate aminotransferase, (**J**) alanine aminotransferase, (**K**) lactate dehydrogenase, and (**L**) alkaline phosphatase. The results are presented as the mean ± 95% confidence intervals, and *p*-values from unpaired two-tailed *t*-tests are expressed as follows: * *p* < 0.05.

The protective effects of GCE treatment on glucose metabolism in *ApoE^-/-^* mice paralleled the beneficial effects on body weight gain and adiposity. As shown in Figure 3A, the effects of GCE on total body weight gain were evident immediately after the diet shifted from chow to the HFD at Week 5. Moreover, after 12 weeks of ad libitum HFD feeding, GCE-treated mice gained 50% less weight than control mice (Figure 3A; repeated measures ANOVA: treatment effect: F_(1, 20)_ = 47.48, *p* < 0.0001). This effect did not seem to be related to toxicity since GCE-treated animals did not show clinical (appearance and behavior) or biochemical alterations, as indicated by circulating markers of liver (Figure 4F–I), kidney, and pancreas function (vehicle vs. GCE: creatinine 0.7 mg/dL (95% CI: 0.1–1.4) vs. 0.6 mg/dL (0.2–0.9), *p* = 0.14; α-amylase 1008.0 U/L (928.1–1089) vs. 889.9 U/L (772.1–1008), *p* = 0.12). Calorie/water intake was also not affected (Figure S1). The lower body weight in the GCE group was accompanied by a significant reduction in the total weight of the epididymal (49%) and perirenal (48%) fat pads (Figure 3B,C). However, adipocytes did not show significant differences in cell size or number (Figure 3D,E).

We also measured levels of molecular markers with the potential to explain weight maintenance, including urine catecholamines and metanephrines, serum leptin, adiponectin, and IGF-1 [55]. We observed that mice treated with GCE displayed lower levels of catecholamines, metanephrines (Figure 3F), and leptin (Figure 3G) than the control animals, while adiponectin (Figure 3H) and IGF-1 levels (Figure 3I) were similar between the two groups.

### 3.4. GCE Improves Nonalcoholic Fatty Liver Disease (NAFLD) and Fibrosis

Representative images from liver sections depicting steatosis and fibrosis are shown in Figure 4A,C, respectively. As expected [54], the control ApoE^-/-^ mice displayed signs of hepatic steatosis and fibrosis in response to the HFD. Interestingly, the GCE-treated mice exhibited reduced hepatic lipid accumulation (Figure 4B) and fibrosis (Figure 4D) and displayed a 16% decrease in liver weight (Figure 4E). In addition, GCE treatment decreased the content of total triglycerides (Figure 4F); specifically, 1-palmitoyl-2,3-distearoylglycerol, 1-palmitoyl-2-oleoyl-3-stearoyl glycerol, and 1,3-distearoyl-2-oleoylglycerol levels were significantly reduced. No changes were observed in hepatic free fatty acids (FFAs) (Figure 4G) or cholesterol (Figure 4H; Table S4).

Regarding the accumulation of extracellular collagen distorting the hepatic architecture, we observed that livers from the GCE-treated mice showed fewer fibrous scars than the control mice and had less pronounced periportal and sinusoidal fibrosis (Figure 4D). No increase in the serum biomarkers of hepatic function, AST and ALT, was observed (Figure 4I,J). GCE treatment tended to lower LDH (Figure 4K) and significantly reduce ALP (Figure 4L). Lower ALP levels have been related to improved secretory activity of the intrahepatic biliary epithelium and a lower risk of hepatic fibrosis [56,57].

### 3.5. In Vivo Protective Effects of GCE are Associated with Immunomodulation

**Figure 5:**
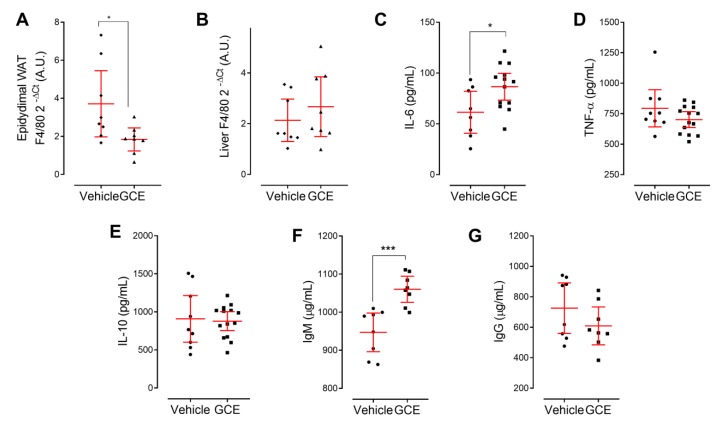
Green coffee extract (GCE) modifies various immune markers in dyslipidemic ApoE^-/-^ mice. The inflammatory status of GCE-treated and control ApoE^-/-^ High-fat diet-fed mice was evaluated via the assessment of immune markers in serum and target organs. The expression levels of (mRNA) for the macrophage marker F4/80 were determined in (**A**) epididymal white adipose tissue (WAT)and (**B**) liver tissues and were expressed as arbitrary units (Arbitrary Units A.U.). (**C**–**E**) Interleukin 6 (IL-6), tumor necrosis factor α (TNF-α), and Interleukin 10 (IL-10) were quantified in liver homogenates by ELISA. Total serum IgM (**F**) and IgG (**G**) levels were quantified by ELISA. The results are shown as the mean ± 95% confidence intervals, and *p*-values from unpaired two-tailed t-tests are expressed as follows: * *p* < 0.05; *** *p* < 0.001.

The immune/inflammatory response is increasingly recognized as a factor that contributes to CMS, with chronic low-grade inflammation as a common factor linking immune and metabolic dysfunction. Immunometabolic dysfunction underlies the initiation and/or progression of insulin resistance/type 2 diabetes, fatty liver disease, and atherosclerosis [58,59]. In an attempt to better understand the protective effects of GCE on metabolic regulation, weight, adiposity management, and hepatic health, we also investigated the magnitude of the inflammatory infiltrates in adipose and hepatic tissue by qPCR. Interestingly, the abundance of F4/80 mRNA in epididymal WAT was lower in the GCE-treated mice than in controls, suggesting reduced macrophage infiltration (Figure 5A). In contrast, despite the hepatoprotective effects of GCE, liver preparations from GCE-treated and control mice had a similar abundance of this marker (Figure 5B). We extended our immunological analysis in liver tissue by quantifying pro-/anti-inflammatory cytokines and found that GCE treatment increased the levels of IL-6 (Figure 5C), whereas the levels of TNF-α and IL-10 remained unchanged (Figure 5D,E). Finally, considering the purported role of B cells in atherosclerosis, obesity, and liver disease [60,61,62], we also investigated the circulating levels of total IgG and IgM antibodies. An increased level of total IgM (Figure 5F), but not of total IgG, was observed (Figure 5G).

### 3.6. GCE Contributed Changes in the Gut Microbiotas

**Figure 6:**
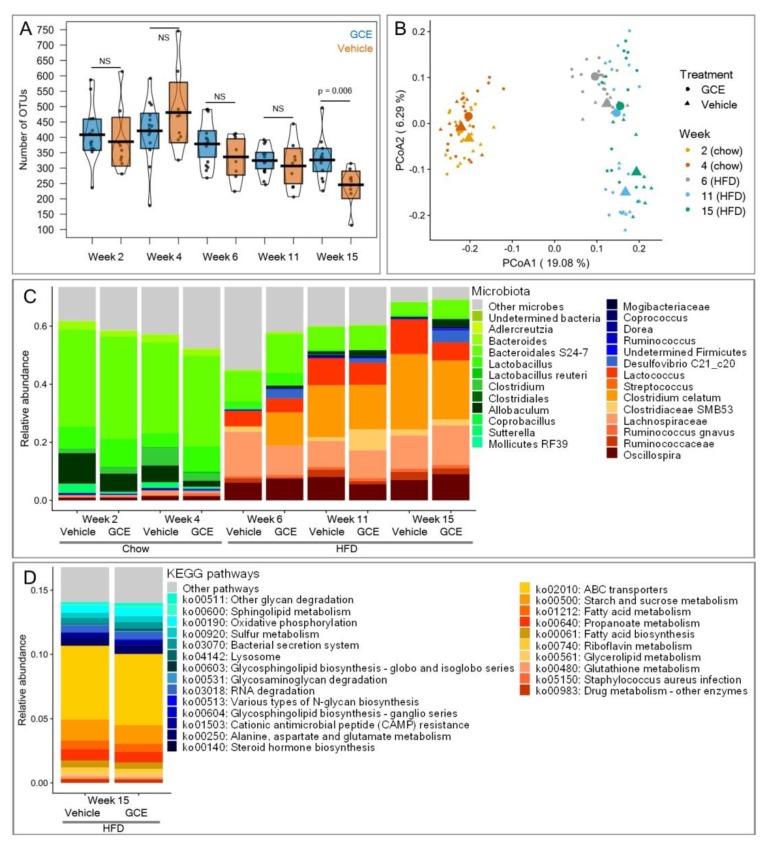
Green coffee extract (GCE) treatment in ApoE^-/-^ high-fat diet (HFD)-fed mice is associated with changes in gut microbiota. (**A**) The number of observed operational taxonomic untis (OTUs, alpha diversity) diminished with time and was significantly higher in the GCE-treated mice (blue boxes) than in the vehicle-treated mice (orange boxes) at the end of experiments (Week 15). (**B**) The chow-to-HFD shift prompted strong modulation of gut microbiota, which was further modified by GCE. Principal coordinates analysis (PCoA) on unweighted UniFrac distances (beta diversity) showed changes in gut microbiota throughout experiments and time. The centroids of each treatment and time point are highlighted with larger points. Percentages on the axes represent the proportion of explained variation of each component of the PCoA. (**C**) Median relative abundance of microbes significantly associated with the chow diet (green tones), the HFD (red tones), or GCE (blue tones). (**D**) Median relative abundance of Kyoto Encyclopedia of Genes and Genomes (KEGG) pathways associated with gut microbiota of mice treated with vehicle (red tones) or GCE (blue tones) at the end of experiments (Week 15).

Gut microbiota can trigger immunometabolic dysfunction, acting as a key contributory factor in CMS. Because the microbiota can be shaped by dietary interventions, we finally assessed the effects of GCE treatment on the microbial community. Both vehicle-treated and GCE-treated mice exhibited significant losses of alpha diversity throughout the experiments (log-likelihood ratio test: chi-squared = 32.8, *p* < 0.0001, Figure 6A), which was likely due to the strong selection of gut microbes imposed by the HFD. The vehicle-treated mice lost 36% of the OTUs in their microbiota at Week 15 compared to the number of OTUs in Week 2. However, GCE treatment significantly alleviated this loss, as animals supplemented with the extract had 25% more microbes at the end of experiments than the control mice (treatment effect at Week 15: F_1,21_ = 9.13, *p* = 0.006, Figure 6A). In terms of beta diversity, in addition to the notable change in gut microbiota that was induced by the chow-to-HFD dietary shift in our experimental setup (PERMANOVA: unweighted UniFrac: *R*^2^ = 0.23, *p* = 0.001; weighted UniFrac: *R*^2^ = 0.54, *p* = 0.001), the GCE treatment contributed additional significant differences (unweighted UniFrac: *R*^2^ = 0.03, *p* = 0.001; weighted UniFrac: *R*^2^ = 0.02, *p* = 0.001) that became more marked as the experiments progressed (unweighted UniFrac: *R*^2^ = 0.034, *p* = 0.001; weighted UniFrac: *R*^2^ = 0.03, *p* = 0.002) (Figure 6B). The changes in gut microbiota were not observed community-wide but affected particular groups of microbes. According to the LEfSe analysis, 12 OTUs were over-represented at Week 2 (chow diet, no GCE supplementation) compared with Week 15. At Week 15, eight OTUs significantly increased in abundance due to the HFD, whereas six were additionally promoted by GCE. The latter included OTUs from *Mogibacteriaceae*, *Coprococcus*, *Dorea*, *Ruminococcus*, *Firmicutes*, and *Desulfovibrio C21_c20* (Figure 6C). Finally, 14 metabolic pathways were significantly more abundant in the microbiome of GCE-treated mice and 10 in the microbiome of vehicle-treated mice at the end of experiments. Of note, pathways related to host glycan degradation and sulfur metabolism were promoted by GCE, whereas pathways related to lipid metabolism, membrane transport, xenobiotics degradation, and bacterial infectious diseases were significantly more abundant in vehicle-treated animals (Figure 6D).

## 4. Discussion

In the present study, we explored the effects of oral supplementation with a water-based GCE on different features of CMS in ApoE^-/-^ mice. Our main findings suggest that, although atherosclerotic plaque and serum lipids did not improve in response to the GCE treatment, it clearly induced several favorable changes in metabolic and immune related markers; specifically, improved metabolic parameters, such as fasting glucose, insulin resistance, serum leptin, urinary catecholamines, and liver triglycerides, were observed. These changes were accompanied by reduced weight gain, decreased adiposity, lower inflammatory infiltrate in adipose tissue, and protection against liver damage. Interestingly, GCE also modulated hepatic IL-6 and total serum IgM, and induced shifts in gut microbiota. Altogether, our results reveal the co-occurrence of all these beneficial cardiometabolic effects in response to GCE treatment in the same experimental model.

Despite the widely accepted hypothesis that dietary polyphenols might protect against atherosclerotic progression [63] and contrary to our expectations, oral treatment with GCE, at a dose equivalent to 220 mg/kg of CGA, three times a week for 14 weeks did not significantly modify atherosclerotic lesions. This result is contradictory to recent results, showing that the treatment with a purified extract from *Lonicera japonica* flowers, almost doubling our CGA dose (400 mg/kg), reduced the area of atherosclerotic lesions by 50% in ApoE^-/-^ mice [53]. Although it was not statistically significant, our extract reduced T-cell infiltration by 20% compared to the vehicle group; a result that should not be overlooked considering that plaque composition plays a pivotal role in the progression of atherosclerosis [58,64]. Thus, despite not reaching statistical significance, the GCE treatment prompted changes in plaque composition related to atheroprotective traits. These observations suggest that further optimization of our extract, by increasing the CGA content for instance, could result in atheroprotective effects, a hypothesis that needs to be confirmed in further experiments.

Our results show that, by reducing weight gain and adiposity and modulating fat inflammation, GCE might compensate for adipose tissue and metabolic dysfunction, thereby blunting central obesity and insulin resistance, which are thought to represent common underlying factors of CMS. Since coffee is a complex mixture containing more than 1000 substances, including CGAs and caffeine [19], it has been reported that some of its beneficial metabolic effects might be mediated, at least partially, by caffeine [65,66,67,68]. Caffeine is completely absorbed in the small intestine and needs around 45 min to become 99% bioavailable [69] and distributed to the tissues where caffeine is known to increase lipolysis and stimulate catecholamine secretion, which can increase energy expenditure [70]. It is finally eliminated from serum and tissues within 4–5 h in urine (95%) [71], after extensive hepatic metabolism by CYP1A2. To rule out the possibility that caffeine was primarily responsible for the positive effects of GCE on body weight and adiposity, we evaluated the urinary concentrations of catecholamines and metanephrines [72]. Catecholamines, particularly epinephrine and norepinephrine, are considered master regulators of lipolysis [73] and are released upon leptin-induced activation of the sympathetic nervous system [74]. Mice that were treated with GCE (the only source of caffeine in our experimental procedures) exhibited lower levels of catecholamines and metanephrines, which is indirect evidence that caffeine was not the main contributor to the observed beneficial metabolic effects. In contrast, vehicle-treated mice not only presented high levels of catecholamines and their metabolites, but also high levels of fasting blood leptin and glucose, findings that are in line with the so-called “leptin and catecholamine resistance state in obesity” [75], which was reversed by GCE treatment. These findings suggest that, in addition to its effects on adiposity and glucose homeostasis, GCE also might be exerting its beneficial effects by increasing sensitization to leptin and catecholamines. Although our current results suggest that GCE might be contributing to adipose tissue homeostasis, at least in part, by controlling HFD-induced inflammation (Figure 5A), adipocytes also play an important role in sensing energy storage in response to anabolic (insulin) and catabolic (leptin or catecholamine) signals [76]. Thus, future experiments to comprehensively explore the potential effects of GCE on adipocytes, particularly those related to adipogenesis, are warranted.

It was previously shown that ApoE^-/-^ mice develop NAFLD/NASH within 7 weeks when fed an HFD enriched with 2% cholesterol [54]. Our results show that longer exposure to a standard HFD containing 0.2% cholesterol also resulted in NAFLD/NASH, since ApoE^-/-^ mice fed an HFD for 12 weeks had significant lipid deposition in the liver, which was accompanied by fibrotic changes and the expression of the macrophage marker F4/80. The GCE treatment contributed to ameliorate the HFD-induced hepatic injury by reducing steatosis and collagen deposition without affecting macrophage infiltration. Current hypotheses propose that NAFLD development is triggered by insulin resistance, which then elicits the onset of second hits such as oxidative stress, inflammation, apoptosis, and autophagy. In fact, insulin sensitizers have been demonstrated to improve the biochemical and histological features of NAFLD [77]. Accordingly, the hepatic protection observed in our study might well be related to the effects of GCE on restoring glucose balance. Although the specific cascade of events leading from NAFLD to NASH is still unclear, some authors have proposed that, in addition to inflammation, the progression is determined by the initiation of a liver fibrotic response [78], while others suggest that metabolic disorders and the resulting altered liver lipid profile could be an early event during the progression of NAFLD to NASH [79]. Since GCE significantly reduced total hepatic triglycerides and ameliorated the fibrotic response, its role in the NAFLD to NASH progression needs to be addressed. Together, our observations are in line with previous work showing that green coffee consumption might contribute to preventing NAFLD progression [80]. These results are highly relevant, considering that NAFLD patients exhibit an increased prevalence of preclinical atherosclerosis compared to individuals without NAFLD. Indeed, cardiovascular disease has been reported as the second most common cause of death in NAFLD patients [81].

In addition to their effects on nutrition and metabolism, natural products are thought to influence immunity. In an HFD context, in addition to preventing metabolic dysregulation and lessening the severity of the NAFLD/NASH phenotype, our GCE treatment also modulated key immunological components that play roles in metabolic regulation. Chronic low-grade inflammation in insulin-sensitive tissues, such as the liver and visceral adipose tissue, is central to obesity-associated insulin resistance, glucose intolerance, and NAFLD progression [59,82]. Macrophage infiltration is a hallmark of obesity-related inflammation [83,84], and reduced macrophage-mediated tissue inflammation is expected to prevent tissue dysfunction. We explored the expression of F4/80 in liver and epididymal fat and found that mRNA levels of this macrophage marker were significantly reduced in the adipose tissue but not in the livers of GCE-treated animals. We also observed increased expression levels of hepatic IL-6 in the GCE-treated mice. There is growing evidence showing that IL-6 is required for the proper control of energy expenditure, metabolic functions, and liver regeneration. Persistent IL-6 gene expression can induce a significant reduction in body weight without affecting food intake [85,86,87]. Furthermore, hepatic IL-6 has been shown to regulate glucose metabolism by increasing insulin sensitivity and negatively regulating hepatic glucose release to the periphery, maintaining liver tissue homeostasis and protecting against the progression of steatosis [88]. In fact, modulation of the IL-6/STAT3 hepatic signaling pathway has been proposed as the underlying mechanism accounting for the beneficial effects of Pu-erh tea extract on HFD-induced NASH and insulin resistance [89]. IL-6 also contributes to counterbalance obesity-associated inflammation by favoring macrophage polarization towards the M2 phenotype, both in the liver and adipose tissue [90]. Interestingly, despite the hepatic protective effect of GCE, no changes in the expression of F4/80 were observed in the liver, suggesting that the effects of GCE on liver macrophages might be qualitative rather than quantitative. Future experiments will address whether GCE treatment can modulate macrophage polarization under HFD conditions. Thus, the increased levels of hepatic IL-6 observed in animals treated with GCE might be linked to the amelioration of HFD-induced liver injury and increased insulin sensitivity observed in this group.

Beyond macrophages and T cells, emerging evidence suggests that B cells also contribute to the modulation of obesity-induced adipose tissue inflammation and insulin resistance [91]. Recent studies have described specific subsets of these cells cardiometabolic protective. In the context of obesity, B-1 cells have been shown to attenuate insulin resistance via IL-10 and polyclonal IgM production [61,91,92]. IgM antibodies can interact with oxidation-specific epitopes (OSE), which are danger-associated molecular patterns (DAMPs) that accompany chronic inflammatory processes. In fact, low levels of OSE-specific IgM antibodies have been reported in patients with NAFLD and have been associated with an increased risk for myocardial infarction [60,93]. In line with these observations, we found significantly higher levels of total serum IgM in animals supplemented with GCE. Since obesity/hyperlipidemia have been proposed as causal factors for reduced IgM levels [60], interventions modulating these factors might contribute to restoring the IgM pool of antibodies.

Dietary modifications, in particular high-fat diets, have been linked to gut microbiota dysbiosis [94], which consequently impacts metabolic disorders. In addition to the beneficial metabolic and immunological effects discussed above, our GCE also ameliorated features of HFD-induced dysbiosis. GCE mitigated the loss of microbial richness, promoted the increase in potentially beneficial microbes, and controlled the abundance of disease-associated metabolic functions. Alpha diversity is a crucial parameter linking gut microbiota and health. A healthy microbiota is characterized by high microbial diversity, which is presumed to reflect ecosystem stability and resilience [31,95]. In contrast, low alpha diversity has been linked to pathological conditions, including obesity [96], inflammatory bowel disease [97], and pathogen infection [98]. In our experiments, an HFD was associated with a 36% reduction in alpha diversity, while GCE partly alleviated this loss. However, GCE not only contributed to maintaining microbial richness; it also altered the composition of the microbial community beyond the changes induced by the HFD. At Week 2, when animals were maintained on a chow diet and received no GCE supplementation, the most abundant microbes were *Bacteroidales S24-7* and *Lactobacillus*, bacteria that dominate the mouse gut microbiota [99,100]. These groups were progressively lost with the HFD and were replaced by other microbes (Figure 6C). Among the latter, *Lactococcus* and *Lachnospiraceae* have been shown increased in studies with mice treated with HFDs [101,102], while *Streptococcus* and *Clostridiaceae SMB53* are increased in human individuals with obesity and metabolic dysregulation [103]. These changes were consistent with enrichment in specific metabolic pathways. Of note, the observed enrichment in ABC transporters and pathways associated to lipid metabolism in mice treated with the vehicle is consistent with human findings showing significant over-representation of these pathways in patients with atherosclerotic cardiovascular disease, cirrhosis, obesity, and type-2 diabetes [32]. Interestingly, GCE contributed further changes in gut microbiota, prompting enrichment of beneficial microbes, including *Desulfovibrio*, a sulfate-reducing bacterium that exerts anti-inflammatory effects [104] and that has been linked to liver detoxification [105], and *Mogibacteriaceae*, bacteria negatively associated with the risk of thrombosis [106]. These changes in gut microbiota composition were accompanied by enrichment in metabolic pathways, including sulfur metabolism, host glycan degradation, and antimicrobial peptide resistance, all of which are associated with healthy states in humans [32,107].

## 5. Conclusions

Collectively, our results indicate that our water-based GCE exerted key beneficial effects associated with CMS. In addition, we explored some mechanisms by which the extract might be acting, namely, the modulation of the immunological components involved in metabolic regulation and the alleviation of HFD-induced gut microbiota alteration. Additional studies aiming to expand our current knowledge on how GCE mediates these effects are warranted. As new insights regarding the functional and protective actions of GCE on CMS are still emerging, studies involving human subjects are necessary to confirm these findings.

## Figures and Tables

**Table 1:** Chemical characterization of the green coffee extract (GCE) used in this study.

Composition	mg/g		mg/g
Total carbohydrates	620	Ash	147
Soluble dietary fiber precipitable	<0.136	Calcium	0.635
Low molecular weight soluble dietary fiber	<0.0183	Copper	0.0176
Insoluble dietary fiber	<0.136	Iron	0.0265
High molecular weight soluble dietary fiber	<0.136	Magnesium	4.02
Total soluble dietary fiber	<0.136	Manganese	0.0119
Total dietary fiber	<0.136	Phosphorous	2.45
Crude protein	242	Potassium	67.8
Total cis-unsaturated fatty acids	0.00007	Zinc	0.0416
Polyunsaturated fatty acids	0.00007		
Omega-6 fatty acids	0.00007	Aflatoxin B1	<0.00000909
Cholesterol	<0.182	Aflatoxin B2	<0.00000909
Caffeine	88.9	Aflatoxin G1	<0.00000909
Total chlorogenic acids	159.7 ± 5.01	Aflatoxin G2	<0.00000909
3,4-dicaffeoylquinic acid	1.10 ± 0.00	Ochratoxin A	<0.000018
3,5-dicaffeoylquinic acid	73.06 ± 2.58	Zearalenone	<0.000182
3-O-caffeoylquinic acid	0.06 ± 0.01	Acrylamide	0.0000956
4,5-dicaffeoylquinic acid	0.58 ± 0.00		
4-O-caffeoylquinic acid	47.84 ± 2.24	Calories	
5-O-caffeoylquinic acid	36.74 ± 2.59	3.42 kcal/g	
Caffeic acid	0.18 ± 0.02		

**Table 2:** Blood chemistry and glucose metabolism parameters and indices in GCE- and vehicle-treated ApoE^-/-^ mice.

Parameter	Vehicle	GCE	*p*-Value
Total cholesterol (mg/dL)	923.0 (688.0–1158.0)	839.3 (687.3–991.3	0.49
HDL (mg(dL)	169.1 (99.5–238.7)	142.1 (92.1–192.1)	0.79
LDL (mg(dL)	390.9 (226.8–555.0)	310.7 (199.4–422.0)	0.53
Triglycerides (mg/dL)	173.7 (140.9–206.5)	176.6 (137.7–215.6)	0.81
Glucose (mg/dL)	297.3 (252.8–341.8)	227.1 (184.9–269.3)	0.02
Insulin (ng/ml)	0.54 (0.27–0.80)	0.43 (0.21–0.65)	0.49
HOMA-IR	11.49 (5.29–17.69)	4.61 (2.87–6.34)	0.004
HOMA-B	23.57 (10.63–36.51)	40.77 (10.76–70.78)	0.24
QUICKI	0.28 (0.26–0.30)	0.31 (0.30–0.33)	0.005

Data are presented as the mean ± 95% CI.

## Supplementary Materials

The following supplementary materials are available online at https://www.mdpi.com/2072-6643/11/3/497/s1. Figure S1: Average intake of calories and water, adjusted for mean body weight and measured over a 20 h window. Table S1: Mass spectral parameters used for catecholamine and metanephrine analysis. Table S2: Histological analysis scores for steatosis and fibrosis in the liver. Table S3: GC/MS conditions for free fatty acids, triglycerides, and total cholesterol in liver samples. Table S4. Triglycerides, free fatty acids, and total cholesterol in liver samples.
