# Supplementary material for: Green Coffee Extract Improves Cardiometabolic Parameters and Modulates Gut Microbiota in High-Fat-Diet-Fed ApoE-/- Mice
Source: Nutrients. 2019 Feb 27;11(3):497. doi: 10.3390/nu11030497 (PMC6470615; doi:10.3390/nu11030497)
Supplement: Supplementary file 1 [file nutrients-11-00497-s001.pdf]

Supplementary material

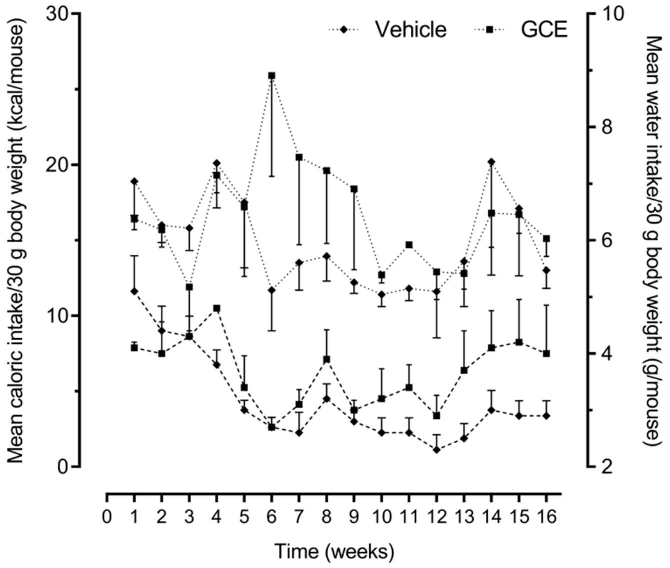

Figure S1. Average intake of calories (dotted line, left Y axis) and water (dashed lines, right Y axis), adjusted for mean body weight and measured over a 20 h window as the difference in the weight between the food/water put into the cage and the remaining food/water at the end of the observation period. Data are expressed as the mean ± SEM.

Table S1. Mass spectral parameters used for catecholamine and metanephrine analyses.

Compound	RT (min)	MRM transitions (m/z)	Cone voltage (V)	Collision energy (V)
Dopamine (u)	1.32	154→91*	22	20
		154→119	22	18
Dopamine-d4 (s)	1.32	158.01→94.72	22	30
3-Methoxytyramine (u)	0.89	168.1→91*	22	24
		168.1→119	22	18
3-Methoxytyramine-d4 (s)	0.89	172.03→94.90	20	34
Norepinephrine (u)	2.12	170→152*	14	6
		170→107	14	20
Norepinephrine-d6 (s)	2.10	176.03→158.07	16	10
Epinephrine (u)	1.54	184.1→166*	20	8
		184.1→107.0	20	20
Epinephrine-d6 (s)	1.54	190.04→172.03	20	18
Normetanephrine (u)	1.24	184.1→166*	20	8
		184.1→134.1	12	18
Normetanephrine-d3 (s)	1.24	187.02→169.07	14	10
Metanephrine (u)	0.97	198.1→180*	18	8
		198.1→165.1	18	18
Metanephrine-d3 (s)	0.97	201.03→183.03	18	16

(u): compounds detected in urine samples (s): spiked at 80 ng/mL in urine samples. *SRM was used for quantification purposes

Table S2. Histological analysis scores for steatosis and fibrosis in the liver.

Lipid content assessment (Oil Red O)			Fibrosis assessment (Picrosirius Red)		
Parameter	***Stage***	***Score***	***Parameter***	***Stage***	***Score***
Vacuolization	Absent	0	Periportal	No fibrosis	0
	Small and scattered	1		Scattered (incomplete lamellae)	1
	Abundant	2		Fibrosis (complete lamellae)	2
				Portal-portal septa	3
Lipid inclusions size	Absent	0	Perisinusoidal	No fibrosis	0
	Microvesicles	1		Mild (5-50%)	1
	Intermediate-vesicles	2		Severe (>50%)	2
	Macrovesicles	3			
Area	< 5%	0			
	25%	1			
	50%	2			
	75%	3			
	>75%	4			

Table S3. GC/MS analysis conditions for free fatty acids, triglycerides and total cholesterol in liver samples

	**Free fatty acids**	**Triglycerides**	**Cholesterol**
Sample preparation:	*50 µL of pyridine + 50 µL of BSTFA 70°C for 2 h, injected directly*	*Dissolved in 100 µL of hexane*	*Dissolved in 100 µL of hexane*
Column:	*Capillary column HP5; 30 m, 0.25 mm i.d., film thickness 0.25 µm, J&W Scientific*	*Capillary column CPTAB Triglyceride analysis; 25 m, 0.25 mm i.d., film thickness 0.1 µm, J&W Scientific*	*Capillary column HP5; 30 m, 0.25 mm i.d., film thickness 0.25 µm, J&W Scientific*
Injection inlet	*280°C, 1 µL*	*380°C, 1 µL*	*280°C, 1 µL*
Oven:	*120°C (5 min), 180°C (7°C/min, 5 min), 200°C (10°C/min, 5 min), 300° (10°C/min, 5 min), 310°C (10°C/min, 5 min)*	*180°C (1 min), 350°C (8°C/min, 1 min), 360°C (2°C/min, 4 min).*	*240°C (1 min), 310°C (15°C/min, 5 min)*
Carrier:	*Helium, 1 mL/min*	Carrier: *Hydrogen, 1 ml /min*	Carrier: *Helium, 1 ml /min*
Detection:	*Mass selective detector (MS) Ionization chamber: 230°C Quadrupole: 150°C 7.40 scan $^{-1}$, 40–700 m/z Ionization energy 70 eV*	*Flame Ionization Detector (FID)*	*MS/SIM ions (Single Ion Monitoring) Ionization chamber: 230°C Quadrupole: 150°C Ionization energy 70 eV SIM Cholesterol Ions: 145 (quantifier)and 275, 255, and 105 (identifier) SIM ISTD ions: 314(quantifier) and 145 and 105 (identifier).*
Quantitation:	*C19:0 standard (5-200 µg/mL) response factor; Signal deconvoluted for identification and quantitation by NIST AMDIS V. 2.68 software*	*Response factors of cocoa butter triglycerides standard (5-30 µg/mL).*	*Cholesterol standard (150-500 µg/mL).*

Table S4. Triglycerides, free fatty acids and total cholesterol in liver samples

Triglycerides (mg/g)	Vehicle	GCE	Free fatty acids and cholesterol (mg/g)	Vehicle	GCE
PPP	0.32 ± 0.15	0.23 ± 0.07	Myristic acid	0.09 ± 0.01	0.10 ± 0.01
MOP	1.62 ± 1.24	0.82 ± 0.64	Pentadecanoic acid	0.03 ± 0.01	0.04 ± 0.00
PPS	6.98 ± 2.76	2.50 ± 1.17*	Palmitoleic acid	0.51 ± 0.15	0.30 ± 0.10
POP	6.66 ± 3.11	6.68 ± 2.67	Palmitic acid	1.45 ± 0.33	1.58 ± 0.21
PLP	1.84 ± 1.41	1.07 ± 0.69	Linoleic acid	0.17 ± 0.04	0.17 ± 0.04
PSS	5.99 ± 7.70	7.70 ± 2.77	Oleic acid	2.62 ± 0.31	2.23 ± 0.36
POS	15.92 ± 10.47	8.84 ± 4.33***	Elaidic acid	0.63 ± 0.69	0.33 ± 0.06
POO	6.20 ± 7.28	0.41 ± 0.24**	Stearic acid	0.37 ± 0.07	0.50 ± 0.08
PLS	1.72 ± 1.04	0.65 ± 0.35	Arachidonic acid	0.14 ± 0.06	0.12 ± 0.05
PLO	0.61 ± 0.22	0.47 ± 0.21	Eicosatrienoic acid	0.03 ± 0.01	0.03 ± 0.01
SOS	34.30 ± 13.83	22.90 ± 10.23**	Eicosadienoic acid	0.07 ± 0.01	0.07 ± 0.01
SOO	3.45 ± 2.86	1.64 ± 0.79	*cis*-11-Eicosenoic acid	0.06 ± 0.01	0.06 ± 0.01
SLS+OOO	5.12 ± 2.81	3.88 ± 1.90	Arachidic acid	0.02 ± 0.01	0.02 ± 0.00
SLO	0.91 ± 0.37	0.87 ± 0.29	Docosahexaenoic acid	0.16 ± 0.15	0.07 ± 0.05
SOA	6.55 ± 3.74	3.84 ± 1.76	Total FFAs	6.36 ± 0.69	5.61 ± 0.67
Total TG	98.82 ± 39.30	66.99 ± 21.66*	Total cholesterol	1.94 ± 0.74	2.05 ± 0.47

PPP: tripalmitin, MOP: 1-margaroyl-2-oleoyl-3-palmitoylglycerol, PPS: 1,2-dipalmitoyl-3-stearoylglycerol, POP: 1,3-dipalmitoyl-2-oleoylglycerol, PLP: 1,3-dipalmitoyl-2-linoleoylglycerol, PSS: 1-palmitoyl-2,3-distearoylglycerol, POS: 1-palmitoyl-2-oleoyl-3-stearoylglycerol, POO: 1-palmitoyl-2,3-dioleoylglycerol, PLS: 1-palmitoyl-2-linoleoyl-3-stearoylglycerol, PLO: 1-palmitoyl-2-linoleoyl-3-oleoylglycerol, SOS: 1,3-distearoyl-2-oleoylglycerol, SOO: 1-stearoyl-2,3-dioleoylglycerol, SLS: 1,3-distearoyl-2-linoleoyl glycerol, OOO: triolein, SLO: 1-stearoyl -2-linoleoyl-3-oleoylglycerol, SOA: 1-stearoyl-2-oleoyl-arachidoylglycerol

*p<0.05; **p<0.01; ***p<0.001
